# The source scaling and seismic productivity of slow slip transients

**DOI:** 10.1126/sciadv.abg9718

**Published:** 2021-08-04

**Authors:** Luigi Passarelli, Paul Antony Selvadurai, Eleonora Rivalta, Sigurjón Jónsson

**Affiliations:** 1 King Abdullah University of Science and Technology (KAUST), Thuwal, Saudi Arabia.; 2 Department of Earth Sciences, University of Geneva, Geneva, Switzerland.; 3 Swiss Seismological Service, ETH-Zürich, Zürich, Switzerland.; 4 GFZ German Research Centre for Geosciences, Telegrafenberg, Potsdam, Germany.; 5 Department of Physics and Astronomy, Alma Mater Studiorum-University of Bologna, Bologna, Italy.

## Abstract

Slow slip events (SSEs) represent a slow faulting process leading to aseismic strain release often accompanied by seismic tremor or earthquake swarms. The larger SSEs last longer and are often associated with intense and energetic tremor activity, suggesting that aseismic slip controls tremor genesis. A similar pattern has been observed for SSEs that trigger earthquake swarms, although no comparative studies exist on the source parameters of SSEs and tremor or earthquake swarms. We analyze the source scaling of SSEs and associated tremor- or swarm-like seismicity through our newly compiled dataset. We find a correlation between the aseismic and seismic moment release indicating that the shallower SSEs produce larger seismic moment release than deeper SSEs. The scaling may arise from the heterogeneous frictional and rheological properties of faults prone to SSEs and is mainly controlled by temperature. Our results indicate that similar physical phenomena govern tremor and earthquake swarms during SSEs.

## Teaser

Aseismic and seismic slip scaling indicate similar physics governing tremor and earthquake swarms during slow slip events.

## INTRODUCTION

Slow slip events (SSEs) are fault ruptures (*1*–*3*) that are too slow to excite detectable seismic waves (*4*). They have been observed in subduction zones (*2*, *5*) and in extensional, transform, and volcanic environments (*6*). Fault zones experiencing SSEs also exhibit other forms of strain release as earthquake swarms [swarmgenic SSEs (SG-SSEs)] (*5*–*7*) and/or clusters of low- and very-low-frequency earthquakes (*8*), embedded in episodic or continuous nonvolcanic tremor [tremorgenic SSEs (TG-SSEs)] (*5*, *9*). These seismic phenomena correlate spatially and temporally with the underlying SSE (*10*), and there is growing evidence to indicate that they can be modulated with the strain rate imposed by the SSE (*7*, *11*–*13*). While geodesy helps to constrain the extent and patterns of slow slip, the seismic fingerprint of SSEs is an indicator of the details of the physical processes associated with large aseismic transients (*14*).

Ordinary earthquakes and/or tremors associated with SSEs are interpreted as localized brittle failure on small-scale asperities triggered by the ongoing aseismic slip front (*7*, *8*). Numerical models (*15*), recent laboratory studies (*16*–*18*), and geological investigations of fault exposures (*19*) support the idea that frictional heterogeneities, variations in the effective normal stress, and variability in shear strength along the fault are responsible for the synchronous presence of large-scale aseismic and localized seismic slip that characterizes SSEs. To our knowledge, no extensive studies have been carried out to investigate the partitioning of seismic and aseismic slip release during SSEs.

Observations of seismic tremor in the Cascadia subduction zone indicate that daily counts of tremor increase proportionally to the size of transient aseismic slip (*13*), and similarly, daily earthquake rates correlate with the aseismic moment rate released during well-documented SG-SSEs (*7*, *20*). Along the Mexican subduction zone, a systematic increase in the magnitude of low-frequency earthquakes (LFEs) coincides with geodetically detected slow slip transients (*21*), indicating that the seismic moment rate of LFEs and the geodetic moment rate of the SSEs are linked by a power law (*11*). This points to a systematic degree of partitioning of the aseismic and seismic moment; when the magnitude of slow slip transients increases, a proportionally larger fraction of seismic energy is released. The LFEs in Mexico exhibit the same interevent time to moment scaling as repeating earthquakes, which is consistent with seismic asperities catching up with the aseismic slip (*11*). The lack of a systematic study of the relationship between seismic and aseismic slip release during SSEs has hampered our understanding of the dynamics of synchronous slow and fast slip.

Here, we compile and analyze a large database of both SG-SSEs and TG-SSEs to investigate the link between the source parameters of aseismic slip and the associated seismic activity. We have identified a robust scaling relationship between seismic and aseismic moment release of SG-SSEs and TG-SSEs. We also examine patterns in duration, hypocenter migration, and rupture velocity and discuss their scaling within the context of previously published scaling behavior for ordinary and slow earthquakes.

## RESULTS

### A database of SSEs and associated seismic activity

**Fig. 1:**
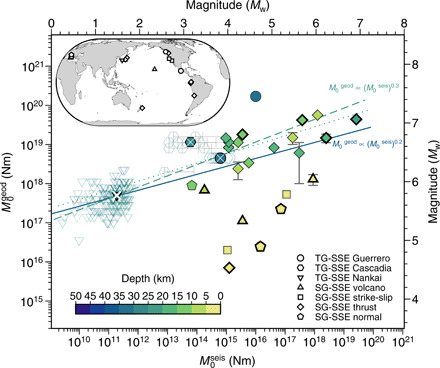
Geodetic moment *M*_0_^geod^ versus cumulative seismic moment *M*_0_^seis^ for TG-SSEs and earthquake SG-SSEs. Symbol shapes indicate the type of data, as shown in the legend. Empty symbols are TG-SSEs, and solid symbols marked with a white cross are the average values. The depth calculated from the centroid of the dislocation model is color-coded. The solid line represents the best-fit nonlinear least-squares regression log(*M*_0_^geod^) = 15.3 (14.9, 15.9) + 0.20 (0.17, 0.24) log(*M*_0_^seis^), with *R*^2^ = 0.4 for all the data. When the seven shallowest SG-SSEs (depth, <5 km) are excluded, the fit (dashed line) becomes log(*M*_0_^geod,*Dp*^) = 14.3 (13.9, 14.7) + 0.29 (0.27, 0.32) log(*M*_0_^seis,*Dp*^), with *R*^2^ = 0.7. Dotted line is the regression fit log(*M*_0_^geod,*Dp*^) = 15.3 (13.3, 17.3) + 0.23 (0.10, 0.35) log(*M*_0_^seis,*Dp*^) using the average values (filled symbols) of TG-SSEs and SG-SSEs. The values in parentheses are the 95% confidence interval. Error bars are the reported uncertainties on moment estimation when available (see Materials and Methods and table S2). The thick outlined symbols mark SG-SSEs with associated off-plane seismic swarms. Inset indicates the geographical location of the TG-SSEs and SG-SSEs (see also fig. S1).

We compiled a new dataset of SSEs that have triggered seismic activity by retrieving data from publicly available databases and directly from the literature. The SG-SSEs and TG-SSEs were selected by considering the seismic activity during the ongoing aseismic slip phase. The collected SG-SSEs occurred in a variety of different tectonic settings (Fig. 1 and fig. S1) and exhibited no seismic tremor at the time of the aseismic slip. The selected TG-SSEs are from subduction zones in North America, Mexico, and Japan, where transient aseismic slip has been associated with enhanced tremor activity (Fig. 1 and fig. S1).

For the database, we separated source properties linked to aseismic and seismic moment release as follows: (i) geodetic moment, *M*_0_^geod^ (i.e., total moment seismic plus aseismic, as constrained by inversion of ground deformation data); duration of the aseismic moment release, *T_G_*; and rupture velocity, *v*_rpt_, defined as rupture length divided by *T_G_*; (ii) cumulative moment released seismically, *M*_0_^seis^; total duration of the seismicity, *T_S_*; and hypocenter migration velocity, *v*_mig_. To determine the characteristic depth of each SSE, we took the centroid of the dislocation model from the geodetic inversion of the aseismic slip (*6*).

The TG-SSEs along the Cascadia subduction zone were compiled from 10 episodic tremor and slip (ETS) events [moment magnitude (*M*_w_) = 6.2 to 6.8] between 1998 and 2009, with each event *M*_0_^geod^ inverted from GPS data and the *M*_0_^seis^ estimated from the associated tremor (*22*). The eight TG-SSEs that occurred in the Guerrero subduction zone (2005–2006 *M*_w_ = 6.4 to 7.5; table S1) also had their *M*_0_^geod^ estimated from GPS data and the *M*_0_^seis^ based on the LFEs’ magnitudes (*11*, *21*, *23*). For the Nankai subduction, we selected 174 TG-SSEs (*M*_w_ = 5 to 6.2) from the Slow Earthquake Database (*24*) that occurred between 2004 and 2015. Source parameters *M*_0_^geod^, *T_G_*, and *v*_rpt_ of these TG-SSEs were derived from inversion of tiltmeter data (see Materials and Methods). We calculated *M*_0_^seis^, *T_S_*, and *v*_mig_ for the LFE activity associated with each of these TG-SSE using the LFE catalog of Japan (*25*). The LFE activity was considered to be associated with an SSE if a burst of LFEs occurred in the time window *T_G_* of the aseismic slip and within an area determined by two fault lengths and widths of the SSE fault model (see Materials and Methods and fig. S2, A and B). We calculated *M*_0_^seis^ as the sum of the seismic moment of the LFEs in each burst using standard moment-magnitude scaling (see Materials and Methods). The estimation of *M*_0_^seis^ is not critically sensitive to the size of LFE selection area; varying it between one and three fault lengths only results in changes of *M*_0_^seis^ bounded within 0.2 to 1.9, with respect to the reference *M*_0_^seis^ (see Materials and Methods and fig. S2C). This verifies that the LFE triggering is a process localized to the aseismic slip with hypocenters distributed up- and down-dip on the SSE fault plane (figs. S2A and S3B). Only 11 of the 174 TG-SSEs along the Nankai segment were not accompanied by synchronous LFE activity (fig. S2). LFE bursts typically have a shorter duration than the associated aseismic release (i.e., usually *T_S_* < *T_G_*), although their durations correlate (fig. S3A). Last, a total of 45 of the LFE bursts show a migration along the SSE source fault strike, and for these cases, we estimated the average *v*_mig_ through a least-square fit (see Materials and Methods).

For the SG-SSEs, we collected and analyzed 23 occurrences (*M*_w_ = 4.9 to 7.2) (see Materials and Methods, table S2, and fig. S1), integrating and expanding a previous compilation (*6*) by including events from a variety of tectonic settings. Our final dataset contains three SG-SSEs that occurred in volcanic zones, two on strike-slip faults, three on normal faults, one on an intraplate thrust fault, and the remainder in subduction zones. We only considered swarm seismicity that occurred in the time window of the ongoing aseismic slip, although the spatial distribution of the swarm earthquakes was sometimes off-plane with respect to the SSE rupture plane (Fig. 1 and table S2). For some SG-SSEs, the onset of the swarm activity was delayed by days with respect to the start of slow slip (*7*), while for other SG-SSEs, the swarms were active throughout the duration of the event (*20*, *26*). However, for many SG-SSEs, no information was available on the duration of the associated seismic swarm (table S2).

### Source scaling of TG-SSEs and SG-SSEs

We find that, on a log-space plot, the relationship between *M*_0_^geod^ and *M*_0_^seis^ for both SG-SSEs and TG-SSEs follows a linear trend across several orders of magnitude (Fig. 1). The SG-SSEs are shallower and accompanied by a larger fraction of *M*_0_^seis^ release when compared to the TG-SSEs with comparable *M*_0_^geod^ (Fig. 1). The seven shallowest SG-SSEs, with a characteristic depth of <5 km, depart from the general trend and align on a steeper scaling. However, in general, neither moment shows an obvious correlation with depth (fig. S4, A and B).

We model the trend in log-space using the scaling relationship *M*_0_^geod^ = β (*M*_0_^seis^)^α^ (Eq. 1). The exponent α modulates the proportion of seismic moment release associated to the total moment geodetically detected, and if α = 1, the scaling becomes linear. Fitting Eq. 1 to all the data returns a statistically significant exponent of α ≈ 0.2 (*p* ≪ 0.01), which covers *R*^2^ = 40% of the data variance. If we exclude the seven SG-SSEs that are shallower than 5 km, the fit can explain 70% of the data variance and the exponent becomes ≈0.3 (Fig. 1). The same scaling results are obtained when, instead of the total moment *M*_0_^geod^, we use the aseismic moment as *M*_0_^aseis^ = *M*_0_^geod^ − *M*_0_^seis^ (fig. S5).

The power-law scaling is not biased by the larger proportion of TG-SSEs, as this regression also fits subsets of data with an equal number of TG-SSEs and SG-SSEs drawn from 1000 Monte Carlo simulations, robustly returning the same fit parameters as in Fig. 1 (see Materials and Methods and figs. S6 and S7). The oversampling bias can also be independently investigated considering that *M*_0_^geod^ released by TG-SSEs has a repeating nature (*27*, *28*). We take the average values of *M*_0_^geod^ and *M*_0_^seis^ for each individual subduction zone as representative of its long-term behavior. We replace the individual events with the average values of moments of TG-SSEs in the Nankai and Cascadia subduction zones and the seven *M*_w_ 6.4 of the Guerrero subduction zone in the data fit for Eq. 1 (Fig. 1). The power-law fit returns the same α ≈ 0.2. The fitting performance degrades (*p* = 0.09 and *R*^2^ = 11%), as expected for a smaller sample and the presence of data deviating from the fit (SG-SSEs shallower than 5 km). When the latter are excluded from the regression, the fit to the power-law scaling improves considerably to *p* ≪ 0.01 and *R*^2^ = 46% for α ≈ 0.2. However, this operation of averaging out TG-SSEs for each area filters out the observed variability in the *M*_0_^seis^ of LFE activity, which covers more than two orders of magnitude, and could be revealing of the underlying physical mechanisms, as we discuss later.

To test the robustness of the scaling against the data uncertainties is challenging because our database is not homogeneous and the uncertainties associated with *M*_0_^geod^ and *M*_0_^seis^ are rarely reported. We assign to each *M*_0_^geod^ and *M*_0_^seis^ a relative error of (Δ*M*_0_/*M*_0_) = 15%, which is larger than the few reported errors in our database (Fig. 1). We translate the data uncertainties in the regression fit via 1000 Monte Carlo simulations from normal distributions of the log-transformed moments with the mean of the observed (*M*_0_^geod,seis^)*_i_* and standard deviation (SD) (Δ*M*_0_/*M*_0_)*_i_* = 15%, where index *i* runs through the data. This translates to a range of variations of both moments within 0.2 to 4 times (*M*_0_^geod,seis^)*_i_* (see Materials and Methods). For each simulation, we fit the power-law model in Eq. 1. The resulting distribution of the regression parameters overlaps the regression fit reported in Fig. 1 (fig. S8A). In addition, separate fits on TG-SSEs and SG-SSEs return statistically significant regression parameters (fig. S8B). Further splitting the SG-SSEs into shallower (<5 km) and deeper (>5 km) events produced substantially improved fits but with a large variability of regression parameters due to the smaller sample sizes (fig. S8B). The result of our statistical analysis indicates that the power-law scaling is a robust feature in our data.

**Fig. 2:**
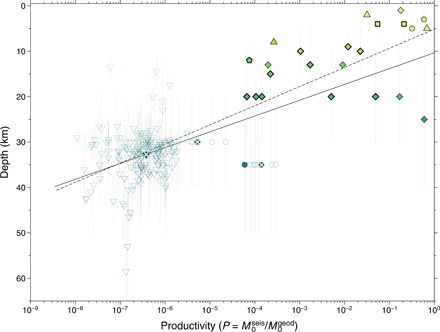
Earthquake productivity (*P*) versus characteristic depth of SSEs. Seismic productivity versus depth of SSEs. Vertical thin lines indicate depth range, and symbols pinpoint the mean value <Depth>. The solid line represents the best-fit regression line <Depth> = 10.3 (6.6, 14.1) − 3.5 (−4.1, −2.9) log(*P*), with *R*^2^ = 0.4 and *P* ≪ 0.01 using all data, while dashed line is <Depth> = 5.1 (−1.8, 12.0) − 4.2 (−6.5, −1.9) log(*P*), with *R*^2^ = 0.4 and *P* ≪ 0.01 using the average value of TG-SSEs. In the regression equations, <Depth> is positive upward. The values in parentheses are the 95% confidence interval.

The ratio *P* = *M*_0_^seis^/*M*_0_^geod^, which we call “seismic productivity” of an SSE, plots over nine orders of magnitude (Fig. 2). *P* quantifies the degree of “brittleness” of the medium hosting the SSE. The data plotted in terms of *P* show a clearer dependence on event depth (Fig. 2) and are consistent with the idea that increasing depth results in diminished seismic productivity (*6*).

**Fig. 3:**
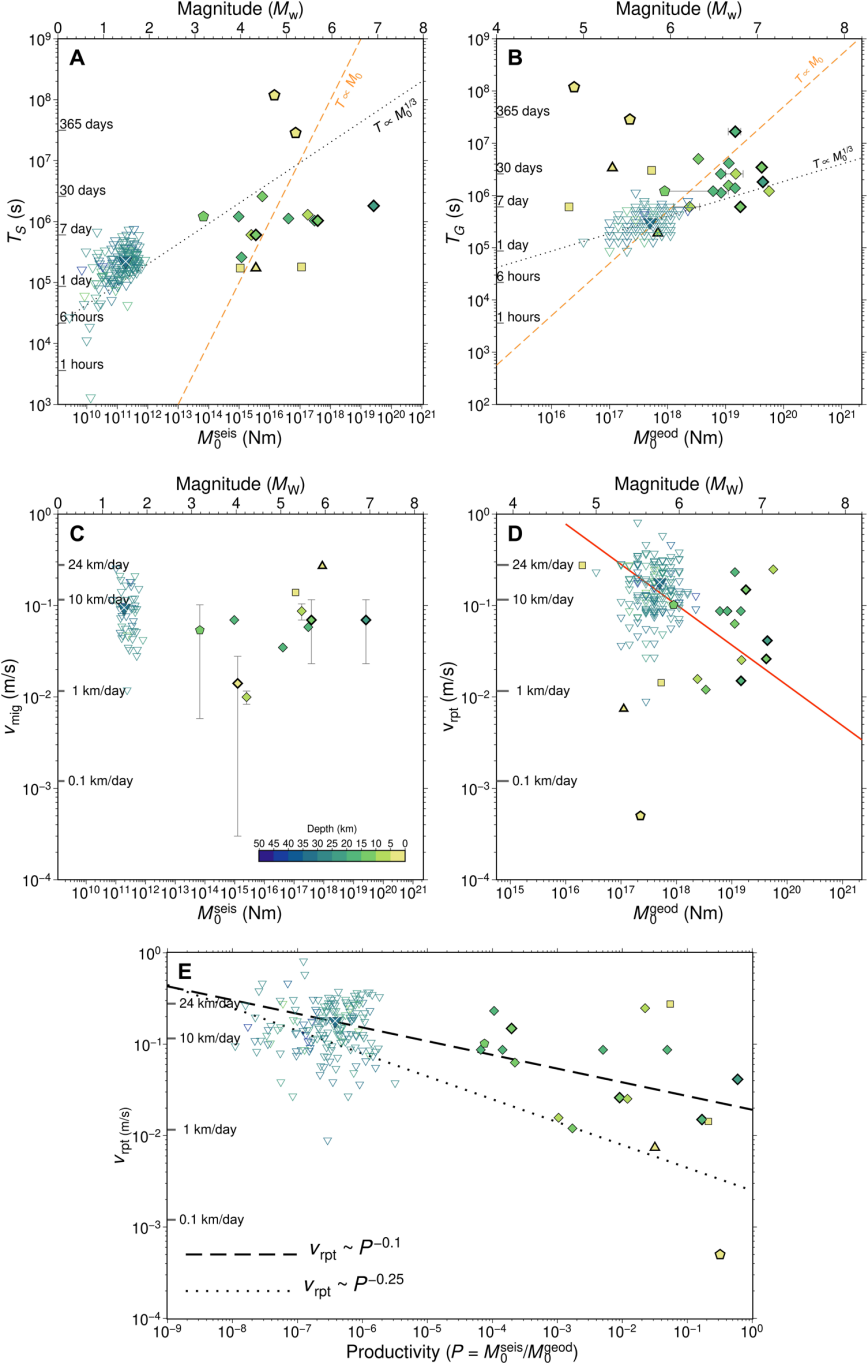
Scaling of durations and velocities versus moments and productivity versus rupture velocity of SSEs. Colors and symbols are as in Fig. 1. (**A**) Seismic duration *T_S_* for SG-SSEs and TG-SSEs versus seismic moment *M*_0_^seis^. (**B**) SSE duration *T_G_* versus geodetic moment *M*_0_^geod^. The lines in both (A) and (B) represent the moment-duration scaling proposed in the literature (*30*). (**C**) Cumulative seismic moment *M*_0_^seis^ versus migration velocity *v*_mig_. (**D**) Geodetic moment *M*_0_^geod^ versus rupture velocity *v*_rpt_. The red line is *v*_rpt_ ~ (*M*_0_^geod^)^−0.5^ (*32*). (**E**) SG-SSE rupture velocity *v*_rpt_ versus earthquake productivity *P*, in comparison with the theoretically derived equation *v*_rpt_ ~ *P*^−γ^ (lines). A least-squares fit to the data returns a statistically significant slope of −0.14 (−0.17, −0.10) with *P* ≪ 0.001 and *R*^2^ = 0.3 and slope −0.2 (−0.38, −0.03) and *P* = 0.02 and *R*^2^ = 0.25 when only the average values of TG-SSEs are used in the regression. The values in parentheses are the 95% confidence interval. Both estimations are within the range of the theoretical values. However, the data show significant scatter around the model indicated by a low coefficient of determination.

*M*_0_^seis^ shows no or weak correlation with the total duration *T_S_* of seismic activity (Fig. 3A), which is in agreement with the recent scaling proposed for tectonic earthquake swarms (*29*), and also does not conform with either a linear (*n* = 1) or cubic (*n* = 3) moment-duration scaling (*M*_0_ α *T^n^*) derived for bounded and unbounded rupture growth (*30*). *M*_0_^geod^, on the other hand, shows a weak scaling with the SSE duration *T_G_* cubed, although the scatter is large and the shallowest SG-SSEs produce outliers (Fig. 3B). Our data are therefore inconclusive for determining whether the *M*_0_*-T* scaling is linear or cubic, but the latter was recently proposed for SSEs in the Cascadia and Mexico subductions (*11*, *31*). However, the characteristic duration seems to be uncoupled from the seismicity, as expected for tectonic earthquake swarms triggered by slow transients (*29*), and conversely, the geodetic duration correlates more closely to the size of the slow deformation.

The seismic migration velocity, *v*_mig_, which ranges from 1 to 24 km/day, shows no correlation to *M*_0_^seis^ (Fig. 3C). A scaling relation from a previous study (*32*), *v*_rpt_ ~ (*M*_0_^geod^)^−δ^, with δ = 0.5 ± 0.1 (Eq. 2) (red line in Fig. 3D), is consistent with our data but with a large scatter of *v*_rpt_ of SG-SSEs (Fig. 3D). Migration velocities (*v*_mig_) are of the same order as the rupture velocities (*v*_rpt_), suggesting that the SSE stressing rate influences the temporal evolution of the seismicity as documented for TG-SSEs (*12*).

The power-law scaling Eq. 1 between the geodetic and seismic moments can be reformulated as earthquake productivity *P* as a function of *M*_0_^geod^; by combining this with Eq. 2, we derive the empirical scaling relationship that relates geodetic rupture velocity to productivity *v*_rpt_ ~ *P*^−γ^, where γ = δ(1 − α)/α. γ is about (0.1, 0.25) (Fig. 3E) for values of α = (0.2 to 0.3) for the fit of Eq. 1 and δ = (0.4, 0.6) from Eq. 2 (*32*). This power-law scaling (Fig. 3E) predicts a decreasing *P* with depth (Fig. 2) and suggests that SSEs with faster rupture speeds release comparatively less strain via seismic activity than SSEs with slower rupture speeds. The large scatter of *v*_rpt_ around the scaling is at least partly due to large uncertainties and trade-offs in geodetic inversions of both moment and finite fault models of the SSEs.

## DISCUSSION

### Subducting asperities and source scaling

The existing power-law scaling between *M*_0_^seis^ and *M*_0_^geod^ (Fig. 1) suggests an interplay between the aseismic and seismic strain release of both TG-SSEs and SG-SSEs. TG-SSEs and SG-SSEs at subduction zones (Fig. 1) have the same scaling, indicating that tremor and seismic swarms originate from a similar mechanism. Geological investigations of exposed faults indicate accommodation of both slow and stable as well as fast and unstable slips on the same or adjacent fault structures (*1*). Changes in pore pressure coupled with pronounced fault roughness may explain such dual frictional behavior (*1*), while temperature is likely a dominant factor on the systematic reduction of earthquake productivity with depth (*6*). It is known that tremor and LFEs release lower seismic energy than earthquake swarms; therefore, the reduction of seismic productivity with depth is not surprising. However, we demonstrate that seismic productivity spans nine orders of magnitude, indicating a variable seismic response to slow slip transients that scales consistently with *M*_0_^geod^ and depth (Figs. 1 and 2). Increase of temperature, smoothness of the interface, and near lithostatic fluid pressure condition are expected to reduce the seismic moment release with depth on asperities. We propose a model to explain the observations of decreasing seismic productivity with depth at subduction zones and discuss the mechanisms of shallowest SG-SSEs that depart from the main trend in Fig. 1.

TG-SSEs in our dataset occur quasi-periodically with similar sizes of *M*_0_^geod^ release. In contrast, SG-SSEs are mostly one-off events on the time scale of the catalog. However, for similar-sized TG-SSEs, the associated *M*_0_^seis^ varies over two or three orders of magnitude (Fig. 1). Similarly, the SG-SSEs in the Boso peninsula SSEs in Japan (fig. S9) have similar geodetic moment, and repeat every few years, while having larger variability in the associated seismicity. SSEs at Mt. Etna and Kilauea volcanoes also have short recurrence time (*6*, *33*). Global and local observations in areas prone to slow slip transients demonstrated multiple occurrences of seismic swarms over decades up to centuries time horizon (*34*, *35*). We argue that the short recurrence time of deeper TG-SSEs (*27*, *28*) can be related to low friction and high strain rate, which both promote shorter “aseismic” cycles, compared with most of the up-dip SG-SSEs. We therefore hypothesize that the nonrepeating nature of SG-SSEs, rather than being an inherent characteristic of SG-SSEs as opposed to TG-SSEs, may be due to a longer recurrence time beyond our observational horizon. In any case, both repeating TG-SSEs and SG-SSEs in our catalog show large variability in the seismic response.

We propose that similarly shaped asperities subjected to comparable stress perturbations during SSEs result in seismic swarms or tremor, simply due to different conditions in the pressure and temperature and thus of depth at subduction zones. This model proposes that an asperity is prone to SG-SSEs in the colder, up-dip sections of faults but will favor TG-SSEs in the hotter down-dip fault environment when subjected to similar stress perturbations from the SSE over both domains. If we assume depth invariance of the SSE stress perturbation, we can investigate differences in the seismic productivity between SG-SSEs and TG-SSEs using the depth-dependent asperity model in a frictional framework. SSE tremor and LFEs are thought to be generated by the rupture of small brittle asperities driven to failure by the surrounding slow slip (*10*). The catalog presented here offers insights into earthquake swarms associated with the shallower SSEs (<40 km) in addition to the more frequently studied tremor/LFEs (>40 km). These two different seismic expressions of SSEs may be linked through a model that unifies the significance of small brittle tremor/LFE asperities in deeper hot environments to those occurring in the colder up-dip sections of fault zones.

**Fig. 4:**
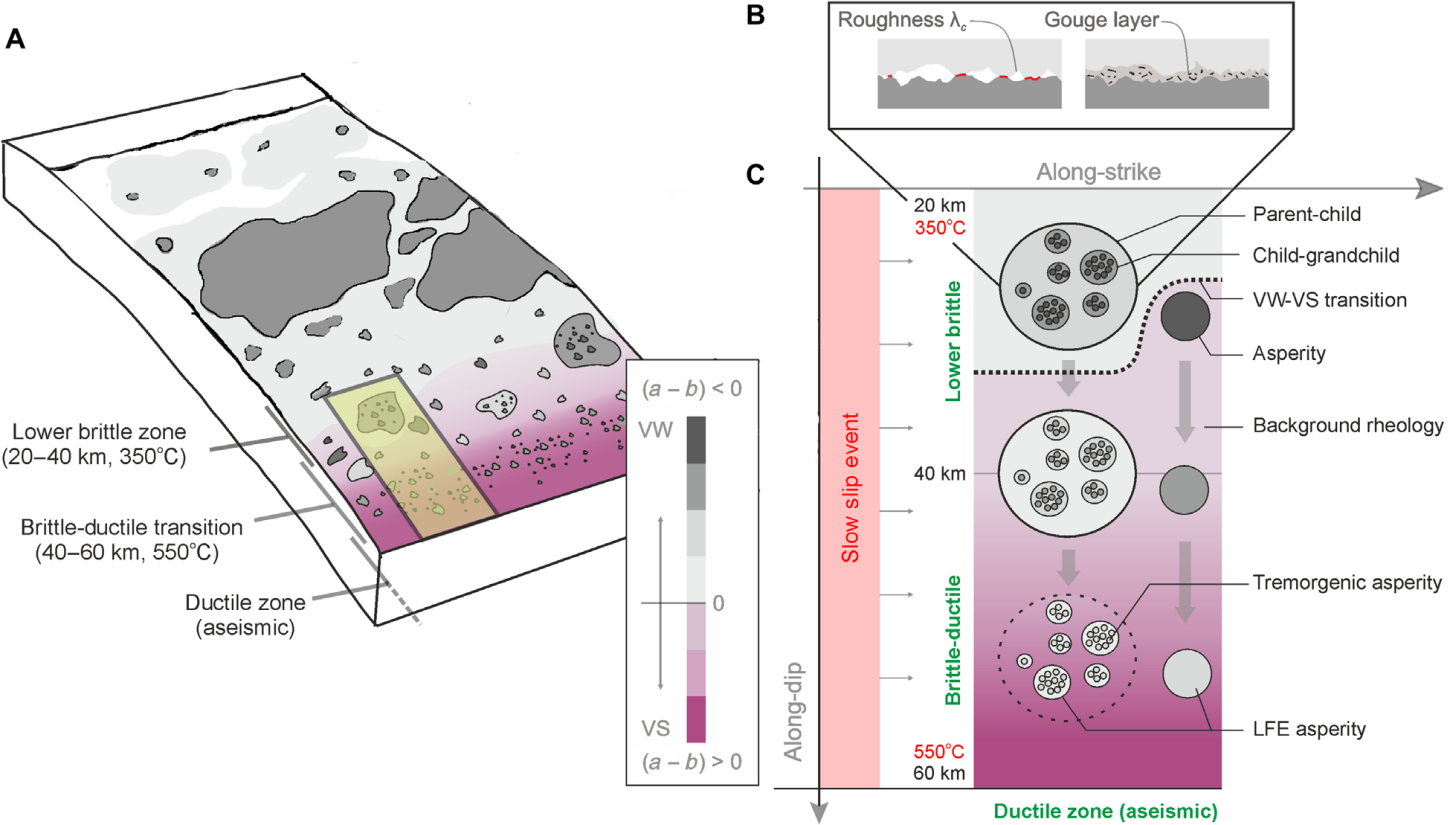
Conceptual model of subducting asperities. (**A**) General schematic of the frictional environment in the megathrust subduction zone based on Lay *et al.*’s model (*36*). The region prone to SG-SSEs and TG-SSEs is highlighted in yellow. (**B**) Mechanisms in the lower brittle zone that can be attributed to heterogeneity and swarmgenic asperities due to variations in critical slip distance *D_c_* associated with wear, the formation of gouge materials, and roughness. (**C**) Subducting asperity model showing the subducting parent-child asperities and their changes with depth. Temperature increases cause VW properties to shift to a VS regime as noted in the laboratory. The level of VW/VS is shown schematically using the color bar and can vary between the asperities and the background.

We propose a model that expands on the depiction of frictional heterogeneity proposed by Lay *et al.* (*36*) and explains the depth-varying rupture properties in subduction zones. Our model adds a layer of frictional complexity to a hypothetical asperity as it subducts from the lower brittle zone (depths of 20 to 40 km) through the brittle-ductile transition (depths of 40 to 60 km). In our model, we propose that additional heterogeneity in frictional properties on a lower brittle zone asperity can produce a swarmgenic response that changes to a tremorgenic response as the asperity subducts. The changes in frictional property in the region highlighted in yellow in Fig. 4A arise from temperature changes from ~350° up to ~550°C as the depth increases (*37*). Laboratory studies have also shown that frictional interfaces can transition from unstable [velocity-weakening (VW)] to stable [velocity-strengthening (VS)] with an increase in temperature past a critical value (*38*). The overall heterogeneity in the background frictional properties—transitioning from VW to VS conditions—is due to this temperature dependence and has been shown to produce SSEs in numerical simulations (*39*).

While large-scale variations in background frictional properties produce aseismic transients, smaller-scale variations within the lower brittle zone (Fig. 4A) have been hypothesized to explain variability in the depth-varying rupture properties of megathrust subduction zones (*36*). Lay *et al.* (*36*) describe heterogeneity in the lower brittle zone as patchy, smaller-scale regions of stable sliding surrounded by conditionally stable areas that transfer to a domain dominated by aseismic slip in the ductile zone. To explain the SG-SSEs in our study, we propose that there is likely variability in frictional heterogeneity on the stable sliding patches in the lower brittle zone. Faults studied in nature, even at shallow depths, show high complexities about the principal slip surface (*40*), suggesting that local variability in frictional properties is likely present. For example, some have hypothesized that there may also be variability in the critical slip parameter *D_c_* (*41*), a parameter that has been attributed to gouge layer thickness between the fault surfaces (*42*) or a critical length scale in the roughness of the interacting fault surfaces (*43*), shown schematically in Fig. 4B. This type of variation could lead to local variations in friction and to what we refer to as a parent-child asperity, depicted in Fig. 4C. Our model proposes that the parent-children configuration of asperities also comprises child-grandchild asperities such that, locally, they exhibit spatial variations in frictional properties. The grandchildren are more prone to seismicity than the children, which, in turn, are more prone to seismicity than the parent asperity, following typical nucleation theory (*44*). While we do not know the precise nature of the frictional heterogeneity (Fig. 4C is for reference only), numerical models that use this parent-child asperity concept can produce earthquake swarms (*45*).

The fate of the parent-child asperity follows that of the surroundings as it subducts into hotter environments. It transitions from a seismogenic asperity in the lower brittle zone (with highly VW properties) to become weakly seismogenic in the brittle-ductile transition (slightly VW properties). It has been noted (*46*) that factors such as fault roughness, gouge content, and pore fluids will affect the temperature dependence of the rate and state friction properties, which can explain why the asperities persist and leave overprinted frictional features that persist as they migrate down-dip. These overprinted small asperities would still exhibit VW properties with respect to the surrounding VS, although the contrast would be lower and their size may be reduced. These sections are the remnant form of the highly seismogenic child-grandchild asperities of the up-dip parent-children, swarmgenic asperity.

Frictional stability is dependent on the critical nucleation length scale h^*^ ~ *D_c_*/[σ_eff_(*b − a*)] (*44*), a parameter that is proportional on *D_c_* and inversely proportional to the VW parameters (*b* − *a*) > 0 and effective normal stress σ_eff_. Numerical simulations (*47*) have shown that the seismic potential of an asperity decreases as the value of h^*^ nears the order of the geometric dimension of the asperity (*L*). It has been demonstrated in laboratory experiments (*48*) that if h^*^/*L* ~ 1, then the fault radiates weak LFE signals. This feature is accounted for in our subducting asperity model, where h^*^ of the daughter asperities in the lower brittle zone is probably smaller due to higher values of (*b − a*) and lower values of *D_c_* (*41*). However, on the remnant, tremorgenic, and LFE asperities, h^*^ would increase due to relatively lower VW (*b − a*) conditions from the temperature increase and the presence of highly pressurized pore fluids that decrease the effective normal stress σ_eff_ (*37*). These features, in combination with the decreasing physical size of the asperities (*L*), would produce weak seismic asperities and explain our observation of lower seismic productivity at these depths.

### Additional physical mechanisms contributing to the source scaling

The unique and depth-dependent scaling of moments for SG-SSEs and TG-SSEs breaks off for SG-SSEs outside subduction zones that have fault centroids confined in the uppermost 5 to 10 km of the crust, as the scaling becomes steeper and the seismic productivity approaches one (Fig. 2). In the colder uppermost crust, the conditions leading to aseismic and seismic slip transients are likely realized through low σ_eff_ rather than a temperature-dependent decrease of the rate and state friction parameters. Local increase of pore fluid pressure, even if at near-hydrostatic level, in a fluid-saturated fault region would promote unclamping and aseismic slip on VS portions of the fault and seismic slip on VW locked asperities. The fact that the seismic productivity approaches one for the shallowest SG-SSEs suggests a similar distribution of VS and VW areas or that VW asperities can propagate beyond the size of asperities. However, for all the events included in this study, the characteristic depths of SSEs in our catalog are not constrained by the distribution of aseismic and seismic slip. Future studies comprising improved SSE depth determination and a larger dataset of SG-SSEs and TG-SSEs will enable a better determination of the uniqueness of this scaling.

The inferred *v*_rpt_ ~ *P*^−γ^ scaling indicates that the rupture speed is inversely proportional to seismic activity, i.e., slower SSEs generate more tremor and swarm seismicity. This observation is counterintuitive for the brittle upper lithosphere, while it may be more representative of those ductile sections at depths of 30 to 50 km and temperatures from 350° to 550°C (Fig. 4), which would more likely produce less seismicity (*49*). The slower rupture velocity of SSEs in shallow fault systems, where earthquake productivity is high, might be linked to increased fault roughness that discourages faster propagation of slow slip rupture. Conversely, fast rupture of SSEs may nucleate in less brittle domains with a lower density of asperities and a more homogeneous distribution of stress and strength, in agreement with the asperity model proposed above. Tsunamigenic earthquakes, for example, are characterized by slow ruptures nucleating in regions characterized by large fault roughness (*50*).

Detailed investigations have shown that the moment rate of SSEs coincides with peaks of seismicity rate (*7*, *11*, *20*), suggesting that the slip rate and thus stressing rate of SSEs modulate the seismicity. Assuming that the maximum slip rate is proportional to the rupture velocity (*51*), *v*_rpt_ in Fig. 3D can be diagnostic of the control of the SSE stressing rate on the seismicity and *v*_mig_ (Fig. 3C). The observation that *v*_mig_ ≈ *v*_rpt_ in Fig. 3 (C and D), together with a lack of clear correlation between *M*_0_^seis^ and *T_S_* (Fig. 3A), supports the hypothesis that the SSE stressing rate plays an additional role in triggering seismic activity.

Well-documented cases of SG-SSEs at subduction zones, transforms, and extensional settings show that the triggered earthquakes are not always coplanar to the SSEs (*20*, *52*, *53*). Examples in our data are indicated in Fig. 1 and fig. S9. This is different from TG-SSEs, where aseismic and seismic slip are thought to be coplanar with the SSEs (*1*). Large SSEs produce transient static stress changes in both large spatial reach and intensity, capable of triggering large-magnitude earthquakes far removed from the rupture plane (*53*). Static stress transfer can thus contribute to the scaling of *M*_0_^seis^ and *M*_0_^geod^ presented in Fig. 1 for SG-SSEs with off-plane seismicity.

In the present work, we built the most up-to-date catalog of SG-SSEs and TG-SSEs where geodetic and seismic source parameters can be compared to investigate synchronous aseismic and seismic slip release. Afterslip and fault creep are processes analogous to SSEs where synchronous release of seismic and aseismic slip occurs. In particular, creeping faults are often characterized by microearthquake activity often in the form of repeating earthquakes (*54*). Recent works suggest that fault creep is not steady state but rather made up of short episodic transients (*55*, *56*). Our database reports shallow aseismic slip transients along the San Andreas and Alto Tiberina creeping faults. In contrast, the aftershock seismicity during afterslip is partly triggered by static stress changes imparted by the mainshock, and it would be difficult to single out the portion of seismicity directly associated with the afterslip. In general, data collected during afterslip would not fit in our simple assumption behind the catalog buildup. Future studies will investigate fault creep and afterslip and their compatibility with the scaling in Fig. 1.

Our proposed model investigates mechanisms underlying aseismic and seismic slip release; however, some examples of slow slip transients are not associated with an increase of seismic activity (*6*). There, the likely frictional properties of the fault are velocity strengthening with negligible density of brittle asperities. As documented here, the occurrence of SG-SSEs and TG-SSEs is generally distinct and spatially separated. However, recent high-resolution seismological investigations along the northern Japan trench highlighted a much more complex intermingling of slow slip transients and the seismic response of the plate boundary fault system (*57*). A rich spectrum of seismic slip release comprising tremor, LFE, and very-low-frequency earthquakes and intermittent swarm activity occurs in segments of the Japanese subduction zone characterized by low coupling and slow slip transients. New and high-resolution studies combining geodesy and seismology are crucial to further investigate the synchronous aseismic and seismic slip release in the context of the scaling law that we derived in this work.

This first global compilation of source parameters of SG-SSEs indicates that both SG-SSEs and TG-SSEs arise from similar mechanisms modulated by depth-dependent frictional conditions. The source scaling implies strong control of the evolution of aseismic slip on the associated seismic response, with the latter decreasing with increasing depth. SG-SSEs and TG-SSEs thus represent a continuum of release of slip on fault systems prone to host silent earthquakes. Future numerical models may be inspired by the scaling law that we derived in this work. The source scaling demonstrated here will help to calibrate theoretical models of SSEs’ rupture propagation and could lead to improved assessments of seismic hazards when the modulation of aseismic slip on the seismic strain release is accounted for.

## MATERIALS AND METHODS

### Characteristics of data of TG-SSEs

Source parameters of SSEs with associated tremor (TG-SSEs) are available through global catalogs, but information on the source parameters of the associated seismic activity is usually poor. Moreover, in many cases, the source parameters of SSEs in TG-SSEs, such as the precise location and finite dislocation model used for geodetic data inversion, are not given in the global compilations or in specific studies. The process of pairing seismic and aseismic source parameters for TG-SSEs also suffers from this lack of uniformity in the reported case studies. The TG-SSEs in this study represent the most updated catalog where both aseismic and seismic source parameters are readily available in the literature. Catalogs of nonvolcanic tremor and LFE events are becoming more and more accessible on the Web. However, when considering tremor, it is not possible to calculate seismic moments, except in particular cases (*22*). The magnitude of LFEs is not routinely estimated; there is an exception, however, such as the LFE catalog published by the Japan Meteorological Agency (JMA) (*25*) and a few specific published case studies (*58*, *59*). A valuable and promising source for slow slip transient processes is the recently published Slow Earthquake Database (http://www-solid.eps.s.u-tokyo.ac.jp/~sloweq/) (*24*), which contains many cases of slow slip phenomena studied using seismological and geodetic investigations. We used this catalog for our database of TG-SSEs by selecting the TG-SSEs for which all the source parameters are available from both deformation and seismological data (fig. S1). Unfortunately, we could not use many examples listed in the database because they lack details regarding the source parameters used in our study, i.e., geodetic moment, *M*_0_^geod^; geodetic duration, *T_G_*; and rupture velocity, *v*_rpt_, defined as rupture length divided by *T_G_*, and, from seismic data, the cumulative moment released seismically, *M*_0_^seis^; total duration of the seismicity, *T_S_*; and the hypocenter migration velocity, *v*_mig_. For the SSEs listed in the Slow Earthquake Database, there is a lack of information on the geometry and dimensions of the finite fault models used in the slip inversion of SSEs. This is an issue that prevented us from using the exceptionally detailed catalog of LFEs in Japan (*25*) with all the SSEs detected in Japan and listed in the Slow Earthquake Database.

Only SSEs along the Nankai subduction, close to Shikoku Island, satisfied our selection requirements. The geodetically inverted SSEs listed the source parameters (location and moment) and finite fault parameters (strike, dip, rake, fault length *L*, and width *W*) for the period 2001–2015, with gaps in 2009 and 2010 (fig. S2). The seismological source parameters associated with SSEs are retrievable from the LFE catalog for Japan produced by the JMA and available through the same database (*25*). The LFE catalog reports the location and magnitude; this is converted to a seismic moment using standard moment-magnitude scaling (*60*). We associate LFE activity to each SSE in time and space as follows: We identified LFEs as associated with an SSE if at least one LFE occurred during the geodetically detected SSE duration *T_G_* and the LFE locations fall within a region that is two times the map-projected fault area [*A_h_* = (2*L*)(2*W_h_*), where *W_h_* = *W* cos(δ), and δ is the SSEs’ fault dip] (fig. S2, A and B). We determined the sensitivity of *M*_0_^seis^ to the size of the LFE search area by using 1*L* or 3*L* (instead of 2*L*) and found that the new *M*_0_^seis^ varies by factors of between 0.2 and 1.9, compared to considering 2*L*, with the bulk of these variations bounded within 0.5 and 1.5 (fig. S2C). *M*_0_^seis^ becomes slightly lower when one fault length is considered, but it is rather invariant when the along-strike distance is increased to 3*L*, indicating that the triggering of the LFEs by the ongoing SSEs is rather localized phenomena along strike of the SSEs. The LFE depth distribution is usually poorly constrained, although it seems to indicate activity distributed around and on the aseismic fault patch (fig. S3). Last, we estimated a first-order along-strike migration of LFEs via a linear least-squares fit. We applied a linear regression to the LFE along-strike coordinate, defined by the SSE plane versus origin time of the LFEs, but only for bursts with more than 20 LFEs. We retained all the regression parameters if the slope was significant, with *p* < 0.001 (*F* test on zero slope). Distances along strike were shifted to the upper left corner of the dislocation plane and time with respect to the first LFEs. The slope of the regression model is thus a first-order estimation of the average migration speed along strike for the bulk of the seismic cloud and therefore can underestimate local variations in the speed of the LFE migration fronts. This methodology identified 45 bursts of LFEs that showed significant along-strike migration.

In addition to the Nankai TG-SSEs, data on TG-SSE source parameters are available for two other locations: the Cascadia and Guerrero subduction zones (Fig. 1). For the Cascadia subduction zone, we obtained data from a published study on the “seismic efficiency” of ETS (*22*). The authors inverted GPS data for 10 ETSs that occurred between 1998 and 2009 with *M*_w_ = 6.2 to 6.8. For each SSE transient, they investigated the associated seismic tremor and calculated the magnitude of the tremor signals. The magnitudes were calculated from the amplitude of the tremor signals and calibrated against local ordinary earthquakes (*22*). The authors suggested that their magnitudes might be underestimated by a factor of 10, which we report as skewed error bars in Fig. 1. Similar estimations of LFE moments for the northern sector of the Cascadia subduction zone are available in the literature (*58*, *61*). The absolute magnitudes reported by Kao *et al.* (*22*) range between *M*_w_ = 0.5 and 2.0 for the Cascadia subduction zone. The seismic moment estimate by Chestler and Creager (*61*) is similar to that of Kao *et al.* and ranges between 1.4 × 10^10^ and 1.9 × 10^12^ Nm (*M*_w_ = 0.7 to 2.1) and is based on LFEs’ displacement recorded underneath the Olympic peninsula. Bostock *et al.* (*58*) derived LFE magnitudes from displacement data rather than waveform amplitudes and obtained moment magnitudes *M*_w_ = 1.0 to 2.6, slightly larger than the other studies (*58*). However, the three studies agree on the magnitude determination within 0.5 magnitude points. The TG-SSE sizes are given as magnitude-equivalent *M*_w_ (*22*); we convert these to *M*_0_^geod^ using the standard moment-magnitude scaling (*60*). The depth of the event is not given by the authors, so we assigned a characteristics depth of 30 km with top and bottom edges between 20 and 40 km, which is the depth range where the ETSs are usually located in the Cascadia subduction zone (*31*).

For the TG-SSEs along the Guerrero segment of the Mexican subduction zone, we used *M*_0_^geod^ and *M*_0_^seis^ provided by W. Frank and published as the moment rate estimate in recent publications (*11*, *21*, *23*) and are reported in table S1. There were eight examples of TG-SSEs that occurred in 2006, of which one had a magnitude of 7.5, while the other events had magnitudes of 6.4. A unique geodetic moment inversion was performed on stacked GPS data of the seven similar transients identified in 2006. The seismic moments were calculated from amplitudes of the LFEs that occurred during each transient (*21*). We assigned 35 km as a characteristic depth, with 20 to 50 km as the bottom-to-top range, in accordance with the locations of the 2006 SSEs in the Guerrero region (*23*).

### Characteristics of data of SG-SSEs

The sources examined and the quantification of the SG-SSE parameters used in this study are detailed in table S2 and reported in fig. S1 with the relative tectonic setting; the information gathered to build up our database is reported below the table for reproducibility. The SG-SSE data are also given as a csv file (SG_SSE_dataset.csv). In general, the data for the source parameters of the SSEs—*M*_0_^geod^, *T_G_*, and *v*_rpt_—were determined from inversions of GPS time series, with the exception of the Pollino (Poll) and Obsidian Buttes fault swarms (Obsi), where InSAR (interferometric synthetic aperture radar) data were also used. If multiple estimations or uncertainties existed for the *M*_0_^geod^ of an event, these values are given in table S2 and indicated with error bars in Fig. 1; the value plotted is the average. Estimations of *M*_0_^geod^ suffer from incomplete or limited spatial coverage of GPS stations; uncertainties associated with the moment inversion are rarely quantified and reported, so we have used a relative error as large as 15% to account for this in our statistical tests. The tendency in many publications is to express *M*_0_^geod^ as the magnitude equivalent *M*_w_, rather than as a geodetic moment. Here, we always converted *M*_w_ to *M*_0_^geod^ using the standard moment-magnitude relation (*60*).

The quantities associated with the earthquake swarms rely on the analysis of the seismicity available in the literature, and these parameters are given in table S2, using seismological data and information with a range of uncertainties. For example, in subduction zones, earthquake catalogs are less accurate and rarely go to *M*_w_ 3 to 4, with Japanese cases being the exception. The instances of SG-SSEs in Italy and the United States are based instead on higher-resolution seismicity catalogs. For this reason, in our analysis, we used quantities such as the cumulative seismic moment *M*_0_^seis^, the total duration *T_S_*, and the average migration velocity of earthquakes *v*_mig_ as representative of the average behavior of the SG-SSEs. In addition, we rely on the assessment of the seismicity provided within the examined sources, which could be subjected to additional uncertainties that we are unable to quantify. Because we have no control of those epistemic uncertainties, we translated the seismic moment *M*_0_^seis^ to the SG-SSE data by considering a large relative error of 15% in our statistical tests. In cases where no information on the cumulative seismic moment was given, we approximated *M*_0_^seis^ using the magnitude of the largest earthquakes; this information is reported in the list at the end of table S2. The general features of the spatial location of seismicity, with respect to the SSE, are given in table S2, e.g., either as in-plane seismicity, when it clearly is coplanar with the aseismic slip, or as off-plane seismicity.

### Data errors in regression fits

We tested the robustness of the two regressions against the uncertainties associated with the values of both moments *M*_0_^geod^ and *M*_0_^seis^ using a Monte Carlo test. As discussed earlier, we considered relative errors in both *M*_0_^geod^ and *M*_0_^seis^ as large as 15% so that Δ*M*_0_^seis^/*M*_0_^seis^ = Δ*M*_0_^geod^/*M*_0_^geod^ = 0.15 and included these errors via the Monte Carlo simulation in the regression. Therefore, we assume that the log-transformed data [i.e., *LM_S,G_* = log(*M*_0_^seis,geod^)] are normally distributed, *N*(μ,σ), with mean μ equal to the *LM^i^_S,G_* and SD σ calculated from the assumed relative errors (Δ*M*_0_^seis^/*M*_0_^seis^)*_i_* = (Δ*M*_0_^geod^/*M*_0_^geod^)*_i_* = 0.15, where *i* indexes the data. If the error propagation formula is applied to *LM_S,G_* as a function of *M*_0_^seis,geod^, it is straightforward to show that the error Δ*LM_S,G_* (Δ*M*_0_^seis^/*M*_0_^seis^)*_i_* = (Δ*M*_0_^geod^/*M*_0_^geod^)*_i_* = 0.15 = σ (*62*), which translates to 1 SD error on the magnitudes of ΔM_W_ ~ 0.05 after applying the standard magnitude-moment scaling (*60*). We performed 1000 Monte Carlo simulations from each of the normal distributions *N*(*LM^i^_S,G_*, 0.15), which allows both moments to vary between 0.2 and 4 times the *M*_0_^seis,geod^, and calculated each slope and intercept of the regression models. The results of the Monte Carlo test are presented in fig. S8A together with the distributions of the regression parameters. The average values and SDs of the regression slopes are α = (0.20 ± 0.01) and log(β_deep_) = (15.38 ± 0.08), where α and β are the parameters of Eq. 1. The distributions of the regression parameters are consistent with the estimation of regression parameters reported in Fig. 1 (insets in fig. S8A), and the regression is significant (*F* test on the regression slope) in 100% of cases. These results indicate that the power-law scaling of *M*_0_^geod^ and *M*_0_^seis^ is a robust feature, including when uncertainties are taken into account.

## SUPPLEMENTARY MATERIALS

Supplementary material for this article is available at http://advances.sciencemag.org/cgi/content/full/7/32/eabg9718/DC1
